# Enhancement in mechanical and antimicrobial properties of epoxidized natural rubber via reactive blending with chlorhexidine gluconate

**DOI:** 10.1038/s41598-023-36962-z

**Published:** 2023-06-20

**Authors:** Thidarat Kanthiya, Nanthicha Thajai, Thanongsak Chaiyaso, Pornchai Rachtanapun, Sarinthip Thanakkasaranee, Anbarasu Kumar, Siwarote Boonrasri, Thorsak Kittikorn, Yuthana Phimolsiripol, Noppol Leksawasdi, Nuttapol Tanadchangsaeng, Kittisak Jantanasakulwong

**Affiliations:** 1 School of Agro-Industry, Faculty of Agro-Industry, Chiang Mai University, Mae Hia, Muang, Chiang Mai Thailand; 2 Nanoscience and Nanotechnology (International Program/Interdisciplinary), Faculty of Science, Chiang Mai University, Chiang Mai, 50200 Thailand; 3 Cluster of Agro Bio-Circular-Green Industry, Faculty of Agro-Industry, Chiang Mai University, Mae Hia, Muang, Chiang Mai Thailand; 4 Department of Rubber and Polymer Technology, Faculty of Engineering and Agro-Industry, Maejo University, Chiang Mai, Thailand; 5 Department of Materials Science and Technology, Faculty of Science, Prince of Songkla University, Songkhla, Thailand; 6 Cluster of Agro Bio-Circular-Green Industry (Agro BCG) and Bioprocess Research Cluster (BRC), School of Agro-Industry, Faculty of Agro-Industry, Chiang Mai University, Chiang Mai, 50100 Thailand; 7 Department of Biotechnology, Periyar Maniammai Institute of Science and Technology, Thanjavur, 613403 India; 8 College of Biomedical Engineering, Rangsit University, Pathumthani, 12000 Thailand

**Keywords:** Biotechnology, Chemistry, Materials science

## Abstract

An epoxidized natural rubber (ENR) blend with chlorhexidine gluconate (CHG) was prepared using a two-roll mill at 130 °C. CHG was added at concentrations of 0.2, 0.5, 1, 2, 5, and 10% (w/w) as an antimicrobial additive. The ENR blend with 10% (w/w) CHG showed the best tensile strength, elastic recovery, and Shore A hardness. The ENR/CHG blend exhibited a smooth fracture surface. The appearance of a new peak in the Fourier transform infrared spectrum confirmed that the amino groups of CHG reacted with the epoxy groups of ENR. The ENR with 10% CHG exhibited an inhibition zone against *Staphylococcus aureus*. The proposed blending improved the mechanical properties, elasticity, morphology, and antimicrobial properties of the ENR.

## Introduction

Currently, the development of renewable biomaterials is an important breakthrough for reducing the environmental impact of synthetic waste. The blending of two or more polymers is a fundamental method for improving the properties of polymers according to their applications. Many biopolymers such as, carboxymethyl cellulose^1–3^, carboxymethyl chitosan^4, 5^, bacterial cellulose^6, 7^, starch^8–12^, gelatin^13^, chitosan^14^, sericin^15, 16^, keratin^17^, pectin^18–20^, polysaccharides^21, 22^, and natural rubber (NR)^23^, have been investigated for the preparation of new biomaterials.

NR is a polymer that is readily available in Thailand and widely used in industry. Epoxidized NR (ENR) is an NR derivative containing an epoxy ring that improves the polarity of rubber and promotes compatibility with other polymers^24–28^. A few studies have used ENR to improve the toughness of polylactic acid^29^. The addition of ENR has been reported to improve the tensile properties and morphology of thermoplastic starch (TPS)^30^. Thermoplastic elastomers are elastic materials obtained by blending a polymer and a rubber^31, 32^. Reactive blending is an effective technique to improve the properties of polymer blends^33–35^. New thermoplastic elastomers have been developed using the ENR reactive melt-blending technique^30^. Crosslinking in the rubber phase is an important reaction for improving the properties of rubbers^36^.

Chlorhexidine gluconate (CHG) is an antimicrobial additive that can be incorporated into rubber^37^. CHG consists of two symmetric 4-chlorophenyl rings and two biguanide groups connected by a central hexamethylene chain that can interact with molecules via hydrogen bonding^38^. CHG can inhibit gram-negative bacteria, gram-positive bacteria, microbacteria, and fungi^39^. Additionally, CHG has been used as an additive in TPS/ENR: it improved the compatibility of the two polymers and increased their hardness^37^. Microbial growth in natural rubber is an important postharvest problem for natural rubber which decreases quality, safety, and properties of rubber material. Addition antimicrobial agent into natural rubber is an effective method to improve antimicrobial properties of material. However, natural rubber is not encapsulated material. Therefore, reactive antimicrobial agent is used to form some reaction and crosslinking in natural rubber to improve mechanical and antimicrobial properties. The natural rubber with antimicrobial properties can prevent microbial growth during bad storage condition and apply for medical application. However, only negligible information is available on the ENR blends with medical fillers that can kill bacteria and fungi and improve mechanical properties in a wide range of industrial uses.

This study new ENR with antimicrobial properties was developed. The objective of this research is to improve ENR properties by blending technique with the antimicrobial compound CHG. CHG was selected due to high antimicrobial properties, heat resistance, and high reactivity by its amino groups of CHG structure. The occurred reaction between CHG and ENR was expected to improve mechanical and antimicrobial properties of ENR. The effects of CHG on the tensile properties, elasticity, hardness, swelling, morphology, reaction, and antimicrobial activity were investigated.

## Methods

### Materials

ENR with 25 mol% epoxidation (ENR25) was obtained from Muangmai Katree Co. Ltd. (Phuket, Thailand). CHG was purchased from S. Tong Chemical Co., Ltd. (Chiang Mai, Thailand).

### Sample preparation

ENR/CHG blends were prepared by melt-blending ENR25 with 0.2, 0.5, 1, 2, 5, and 10% (w/w) CHG using a two-roll mill (Pirom‒Olarn Co. Ltd., Bangkok, Thailand) at 130 °C for 10 min. Low CHG content (lower than 10%) was selected to observed effect of reaction which increased amount of CHG at 2–2.5 time in each condition to find the best condition for mechanical and antimicrobial properties improvement. The as-prepared ENR/CHG blends were then compressed into sheets via hot pressing at 130 °C for 3 min. The codes and compositions of the samples are listed in Table 1.

**Table 1:** Code name and composition of ENR and CHG blends (%w/w).

Samples	ENR	CHG
ENR	100	0
ENR/CHG0.2	99.8	0.2
ENR/CHG0.5	99.5	0.5
ENR/CHG1	99	1
ENR/CHG2	98	2
ENR/CHG5	95	5
ENR/CHG10	90	10

### Tensile properties

The tensile properties were measured following the JIS K 6251-7 standard using a tensile testing machine (Model H1KS, Hounfield test equipment, Surrey, England) at 50 mm/min crosshead speed. The bone-shaped samples were processed into sheets through compression molding at 130 °C for 3 min. The sample dimensions were 2 × 10 × 1 mm (width × gauge length × thickness). Five replicate measurements were performed for each sample.

### Recovery test

The samples were measured for the recovery of elongation as per the JIS K 6251-7 standard using the above-mentioned tensile testing machine. The dimensions of the samples were 2 × 10 × 1 mm (width × gauge length × thickness). The samples were pulled up to 100% and returned to the original position to obtain stress–strain curves at a test speed of 50 mm/min. Five samples were tested under each condition.

### Shore A hardness test

The Shore A hardness of the samples was tested using a Shore durometer (E2-D, Imada Co. Ltd., Toyohashi, Tokyo, Japan). The samples were compressed sheets at 130 °C for 3 min using hot compression molding. Each sample was measured at five positions at room temperature.

### Swelling ratio

Swelling tests were performed according to the ASTM D3616 standard with modification method to use palm oil as solvent due to high swelling index of NR rubber in palm oil^40^. The swelling ratios of the samples were measured by cutting them into 10 × 10 × 1 mm (width × length × thickness) pieces and immersing them in 50 mL palm oil at room temperature for 48 h. The swollen rubber samples were removed from the solvent, and after cleaning the excess palm oil with a paper towel, were accurately weighed using an electronic balance. The swelling ratio was calculated using the following equation^28^.

1 $${\text{Swelling}}\;{\text{percentage }}\left( {\text{\% }} \right){ } = { }\frac{{{\text{W}}_{{\text{s}}} - {\text{W}}_{{\text{u}}} }}{{{\text{W}}_{{\text{u}}} }}{ } \times 100$$

where *W*_u_ and *W*_s_ are the weights of the unswollen and swollen samples, respectively.

### Morphology

Scanning electron microscopy (JSM-IT300LV, Japan) was used to observe the morphological characteristics of the samples at 15 kV. The samples were prepared into 5 × 40 × 1 mm (width × length × thickness) sheets by compression molding at 130 °C for 3 min and broken in liquid nitrogen, followed by sputter-coating of the fracture surface with a thin layer of gold (108 Auto/SE sputter coater, Cressington Co., Ltd., Watford, England).

### Reaction mechanism

The functional groups of the organic species in the ENR/CHG blends were investigated in the 500‒4000 cm^‒1^ spectral range using Fourier transform infrared (FTIR) spectroscopy (FT/IR-4700, Jasco Corp., Tokyo, Japan) with the attenuated total reflectance (ATR) mode. The analyzed thin-film samples were prepared by compression molding at 130 °C for 3 min.

### Antimicrobial activity

The antimicrobial efficacy of the samples against bacterial strains was measured using the agar disc diffusion method. The blended samples were examined against three species of bacteria (*Staphylococcus aureus*, *Escherichia coli*, and *Bacillus cereus*) and three fungal species (*Aspergillus oryzae*, *Rhizopus oligosporus*, and *Saccharomyces cerevisiae*). The reference standards (positive controls) for bacteria and fungi were penicillin and ketoconazole, respectively. The test samples for the agar disc diffusion method were cut into 3 mm size; positive control (10 µL) applied on a 3 mm sterile filter disc and plain filter disc (negative control) were placed on agar plates that were uniformly pre-swabbed with microbial suspensions (equivalent to ~ 107 CFU mL^−1^ from 24 h-grown cultures). Agar plates were incubated for 24 h at 37 °C for microbial growth. The diameter of the inhibition zone was measured to determine its antimicrobial activity. Three replicates were tested for each microbial species.

### Statistical analysis

The test results were analyzed using one-way ANOVA conducted in SPSS software. The differences found (*P* < 0.05) were estimated using Duncan's test.

## Results and discussion

### Reaction mechanism

**Figure 1:**
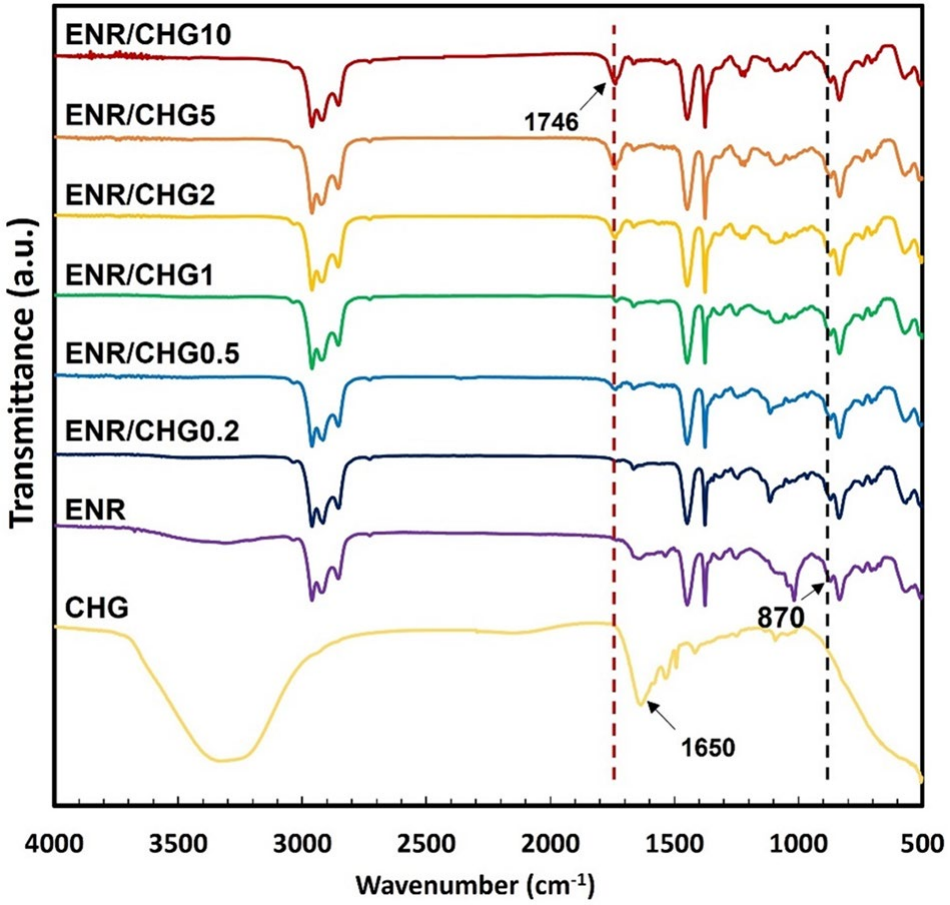
FTIR spectra of CHG, ENR, ENR/CHG0.2, ENR/CHG0.5, ENR/CHG1, ENR/CHG2, ENR/CHG5, and ENR/CHG10 (% w/w).

**Figure 2:**
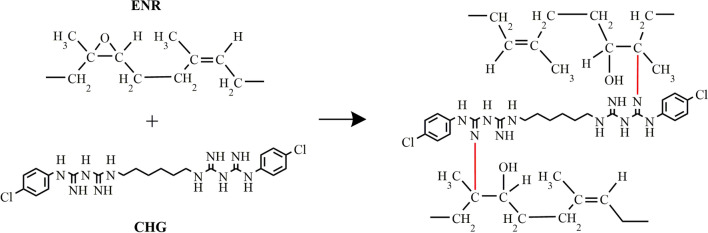
Proposed reaction of the ENR blends with CHG.

Fourier transform infrared (FTIR) spectroscopy was used to observe the reaction between ENR and CHG. The FTIR spectra of the ENR, and rubber composites with varying CHG content are shown in Fig. 1. ENR shows characteristic peaks at 2955 cm^‒1^ (C–H stretching of CH_3_), 2854 cm^‒1^ (C‒H symmetry stretching of CH_2_), 1666 cm^‒1^ (C=C stretching), 1486 cm^‒1^ (C‒H bending of CH_2_), 1382 cm^‒1^ (C–H deformation of the carbon backbone), 1055 cm^‒1^ (C–O stretching of epoxide ring), 870 cm^‒1^ (C‒O‒C stretching of the partial ring opening of the epoxide group), and 835 cm^‒1^ (C = CH wagging)^28^. CHG exhibits characteristic peaks at 1580 cm^‒1^ (stretching of N–H) and 1650 cm^‒1^ (stretching of C–N). Stretching of the chloride-bonded aromatic ring and C–O–C appear at 1095 and 1155 cm^‒1^, respectively^37, 38^. The ENR/CHG blends with CHG at 0.2, 0.5, 1, 2, 5, and 10% (w/w) show a new high-intensity peak at 1746 cm^‒1^, which is not observed in pure ENR. The new peak generated from new vibration of chemical structure in the blend which indicated the occurrence of a reaction during the melt-blending of the ENR/CHG blends. The occurred reaction suggested to epoxy ring opening reaction with amino groups of CHG. This peak, assigned to the new C=O vibration, is a result of the reaction between the epoxy groups of ENR and the amino groups of CHG (Fig. 2). Reaction between epoxy groups with amino groups has also been reported^41–43^. The reaction resulted in the formation of covalent bonds linking ENR with the CHG structure, which improved the mechanical properties of the ENR/CHG blends. The crosslinking in ENR structure formed some network structure via CHG structure while combination effects of ENR network structure and covalent bond from the reaction held CHG on the ENR structure to provide antimicrobial properties.

### Tensile properties

**Figure 3:**
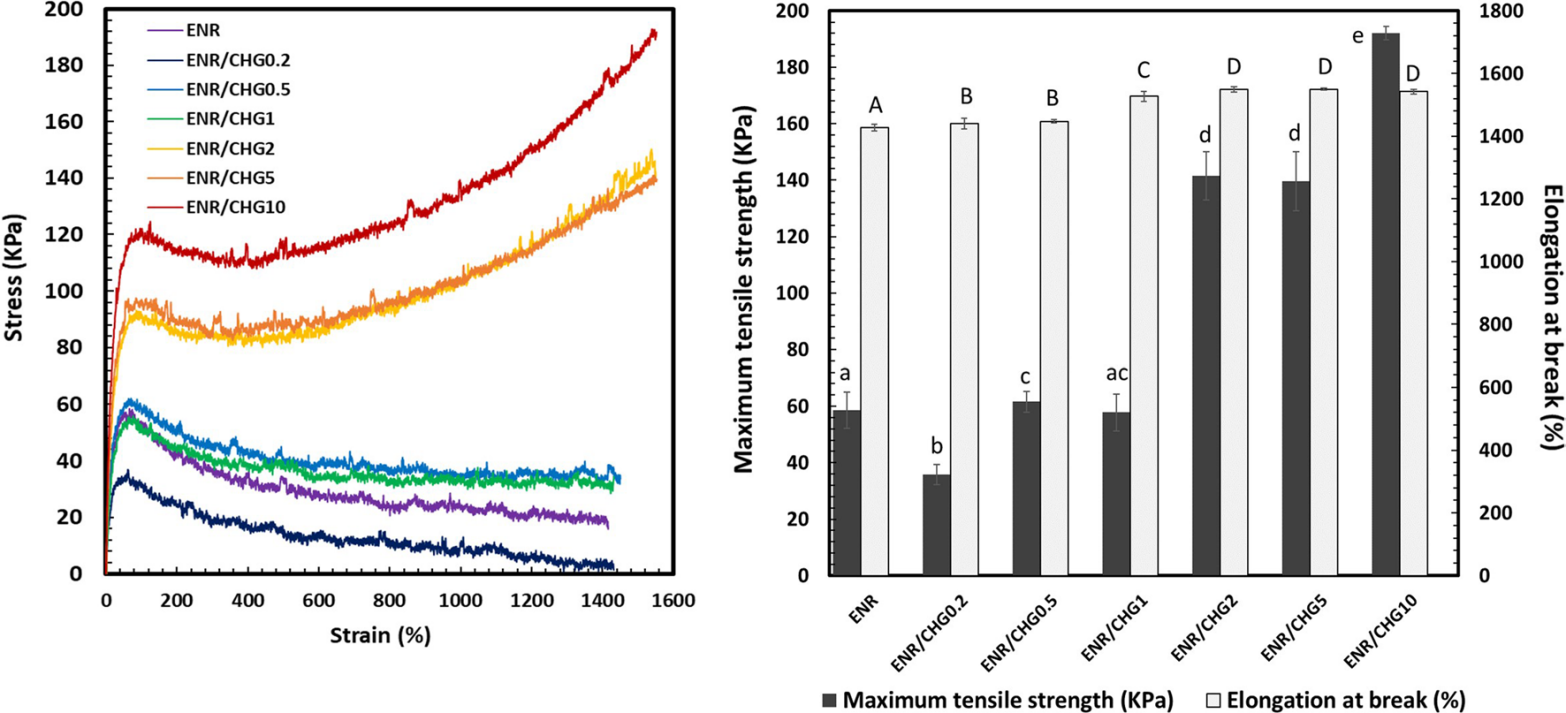
Stress–strain curves of ENR and ENR blends with 0.2, 0.5, 1, 2, 5, and 10% (w/w) CHG, *n* = 5. The mean values of the elongation at break (a–d lowercase letters) and maximum tensile strength (A–D uppercase letters) significantly differ (*P* < 0.05).

The mechanical properties of ENR blended with 0.2, 0.5, 1, 2, 5, and 10% (w/w) CHG are assessed using the stress–strain curve (Fig. 3). The ENR/CHG blend shows higher tensile strength and elongation at break compared with pure ENR. The addition of CHG at 10% increased the tensile strength to 190 kPa, whereas the addition of 0.2% CHG yielded a tensile strength as low as 30 kPa. Increasing of CHG content (0.5–10%) enhanced tensile strength. CHG induced high reaction between CHG and ENR which formed network structure of rubber via the occurred reaction. Improvement in the properties of the ENR/CHG blends (in particular, tensile strength) was achieved through a cross-linking reaction between the epoxy groups of the ENR and the amino groups of CHG. New covalent bonds were formed via CHG as chemical crosslink points and transferred strength through this network structure. Crosslinking with CHG has already been reported to improve the tensile strength of polymer blends^37, 38^.

### Recovery test

**Figure 4:**
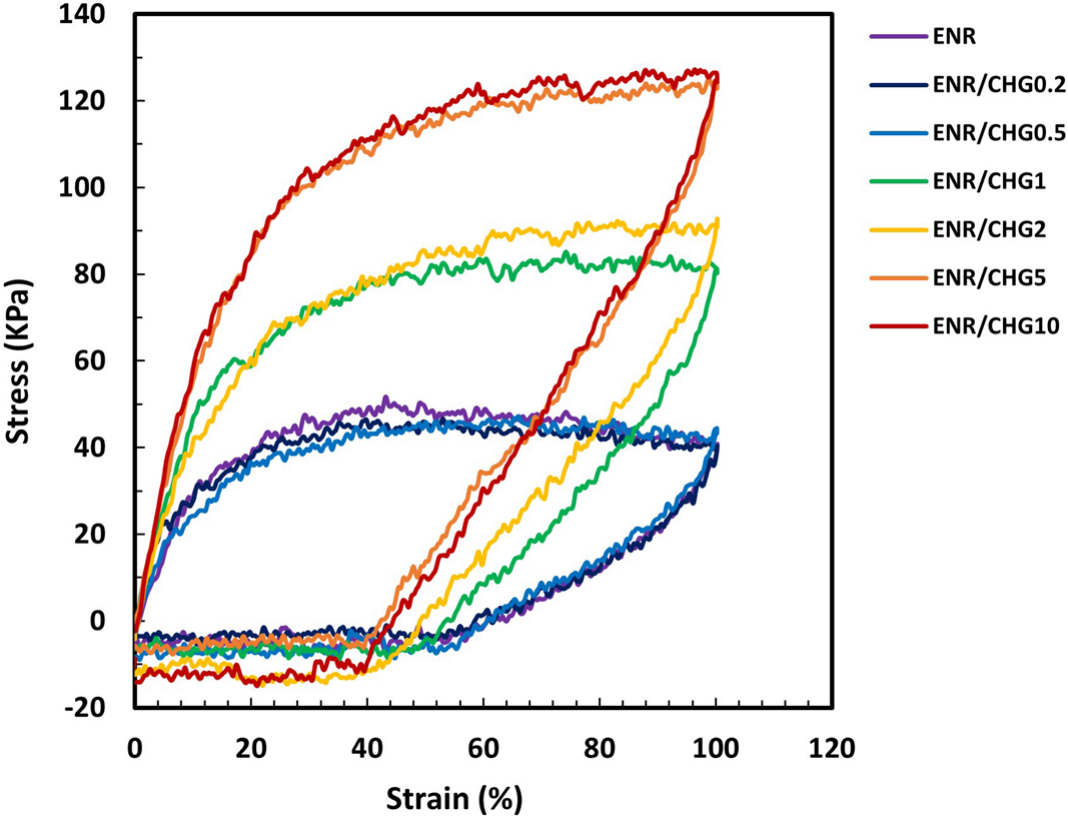
Recovery of ENR and ENR blends with 0.2, 0.5, 1, 2, 5, and 10% (w/w) CHG.

Strain recovery test was performed using tensile tester. The samples were stretched to 100% strain and returned to the original position with the same speed of 50 mm/min. Figure 4 shows the strain recovery of the blended samples. ENR exhibits 50% strain recovery. The high strain recovery of ENR with low tensile strength was due to the absence of ENR chain linkages, whereas mixing at a high temperature (130 °C) resulted in chain scission without crosslinking. Notably, high-temperature mixing of ENR has been previously reported to cause chain scission and induce low tensile properties^44^. These behaviors resulted in the rupture of weak intermolecular bonds in the rubber, causing its inability to completely return to its original shape after deformation^45–47^. The ENR blends with 0.2, 0.5, 1, 2, 5, and 10% CHG exhibit a decrease in the degree of strain recovery. The samples containing 5 and 10% CHG show a strain recovery of 30%, and the samples with 1 and 2% CHG exhibit approximately 45% recovery. The improvements in the stretching and release of ENR/CHG10 indicated an enhanced crosslinking reaction with CHG. This result agrees with a previous report that showed the enhancement of the mechanical properties and elastic recovery of ENR reactive blend with a reaction between the amino group (–NH_2_) and the epoxy group of ENR^37^.

### Shore A hardness

**Figure 5:**
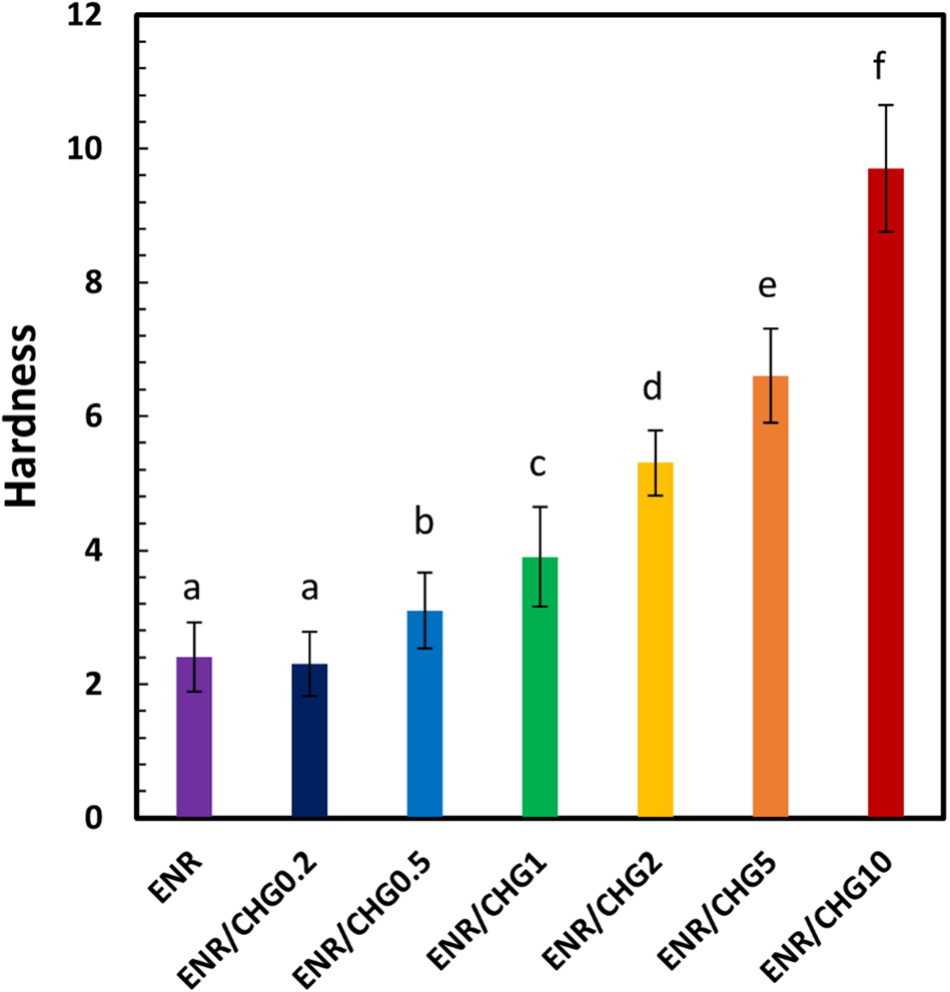
Shore A hardness of ENR and ENR blended with 0.2, 0.5, 1, 2, 5, and 10% (w/w) CHG, *n* = 5. The mean values indicated by the different (a–f) lowercase superscript letters significantly differ (*P* < 0.05).

Figure 5 presents the Shore A hardness of the ENR/CHG blend measured using a durometer test. The Shore A hardness of the ENR is 2, whereas the hardness values of ENR blends with 0.2, 0.5, 1, 2, 5, and 10% (w/w) CHG are 2, 3, 4, 5, 6, and 9, respectively. The blend hardness increased with the increase in CHG content owing to the crosslinking reaction between the amino group (‒NH_2_) of CHG and the epoxy groups of ENR and forming the network structure of the blend. Crosslinking points by the occurred reaction connected rubber structure close together to reduce free volume of rubber structure. High density with low volume fraction of samples by increasing network formation was indicated which increased hardness of the blends. An increase in the hardness of ENR through blending with CHG has been previously reported^38^.

### Swelling ratio

**Figure 6:**
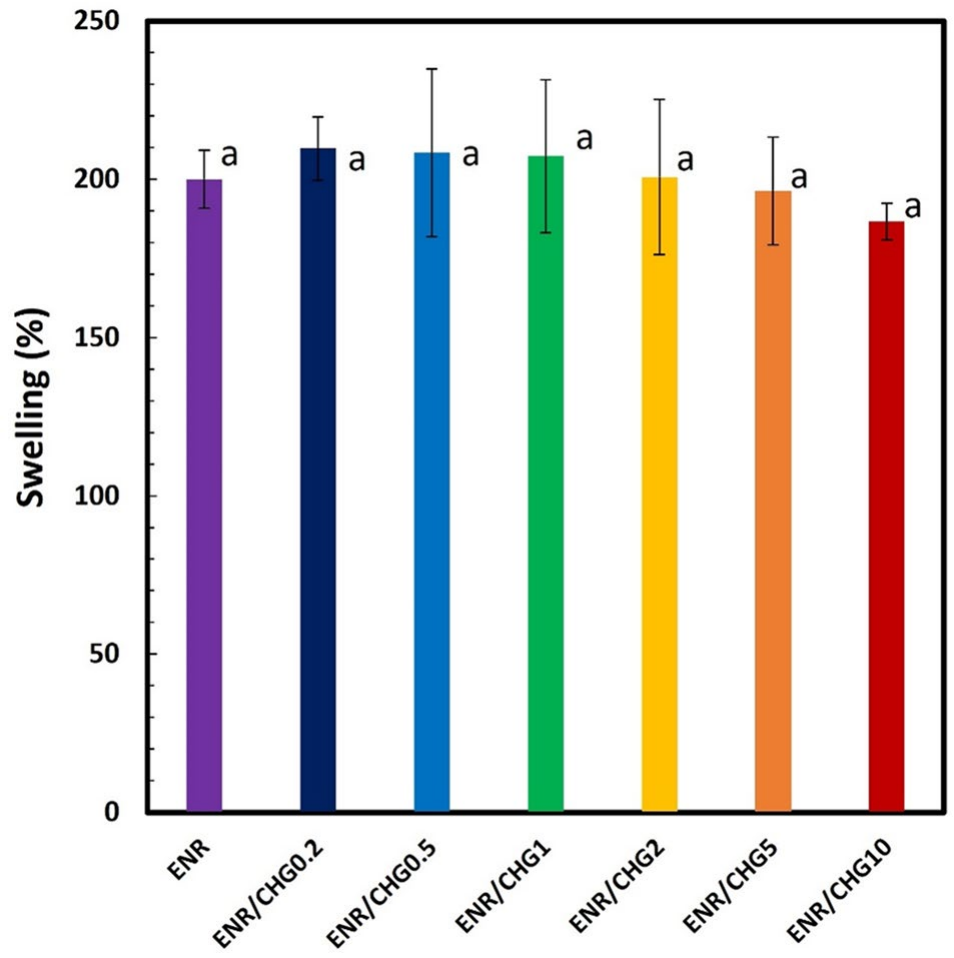
Swelling ratios of ENR and ENR blended with 0.2, 0.5, 1, 2, 5, and 10% (w/w) CHG; *n* = 5. The mean values indicated by the different (a) lowercase superscript letters significantly differ (*P* < 0.05).

The swelling ratio in palm oil was used to assess the crosslinking efficiency of the ENR and ENR/CHG blends. The results of the test are shown in Fig. 6. The swelling ratio of ENR is 200% and those of ENR blended with 0.2, 0.5, 1, 2, 5, and 10% CHG are 209.7, 208.3, 207.2, 200.6, 196.3, and 186.6%, respectively. Low oil swelling with insignificant differences between the samples was attributed to the high oil resistance of ENR due to the polarity of its oxirane groups^48^. Although the results do not significantly change with the concentration of CHG, the swelling ratio of the ENR/CHG10 blend is slightly lower than that of pure ENR. This observation attributed to the strengthening of the network structure via the reaction between ENR and CHG, which resulted in efficient crosslinking inside the rubber. Crosslinking via CHG reaction connected rubber structure close together, reduced chain movement, and decreased oil swelling property of the blend.

### Morphology

**Figure 7:**
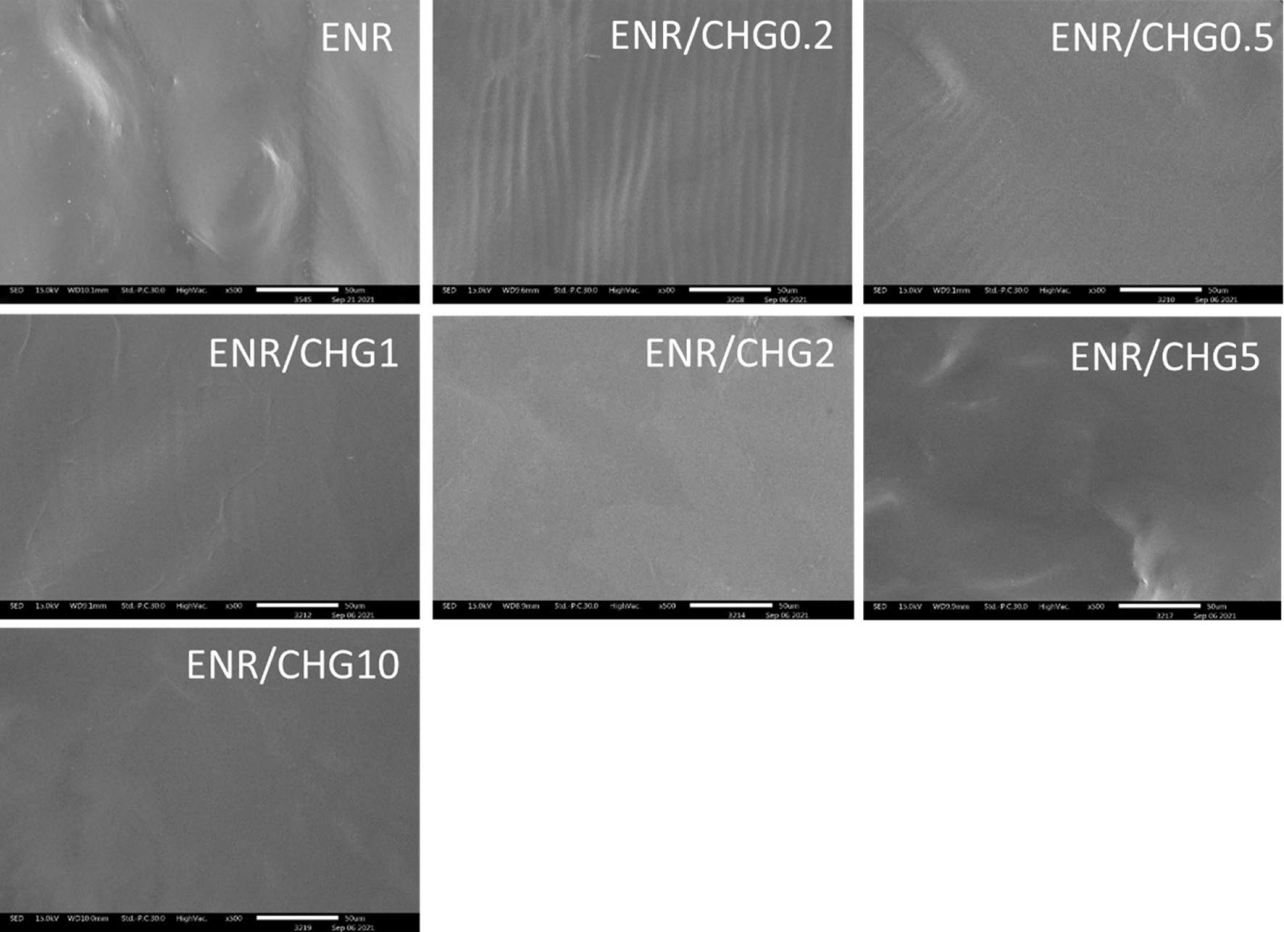
SEM fracture surface images of ENR and ENR blends with 0.2, 0.5, 1, 2, 5, and 10% (w/w) CHG.

The fracture surface morphologies of the ENR and ENR blends with 0.2, 0.5, 1, 2, 5, and 10% CHG observed using scanning electron microscopy (SEM), are shown in Fig. 7. The images of ENR, ENR/CHG0.2, and ENR/CHG0.5 exhibit rough surfaces with slight lines, whereas ENR/CHG1, ENR/CHG2, ENR/CHG5, and ENR/CHG10 blends present smooth fracture surfaces; furthermore, ENR/CHG10 has a high degree of surface smoothness. The lines on the fractured surface of the ENR/CHG0.2 sample indicated a low degree of interconnectedness with the shrinkage of the rubber fracture surface, after breaking in liquid nitrogen. In contrast, samples with high CHG content featured a strong network structure and a smooth fracture surface without shrinkage. A high degree of smoothness was an indication of good miscibility between the ENR and CHG. High CHG content generated many crosslinking points from the occurred reaction which connected ENR structure close together. High density with low volume fraction were indicated, and it provided smooth fracture surface after breaking in liquid nitrogen. The smooth fracture morphology and improvement in mechanical properties attributed to the reaction between the amino groups in CHG and epoxy groups in ENR.

### Antimicrobial activity

**Figure 8:**
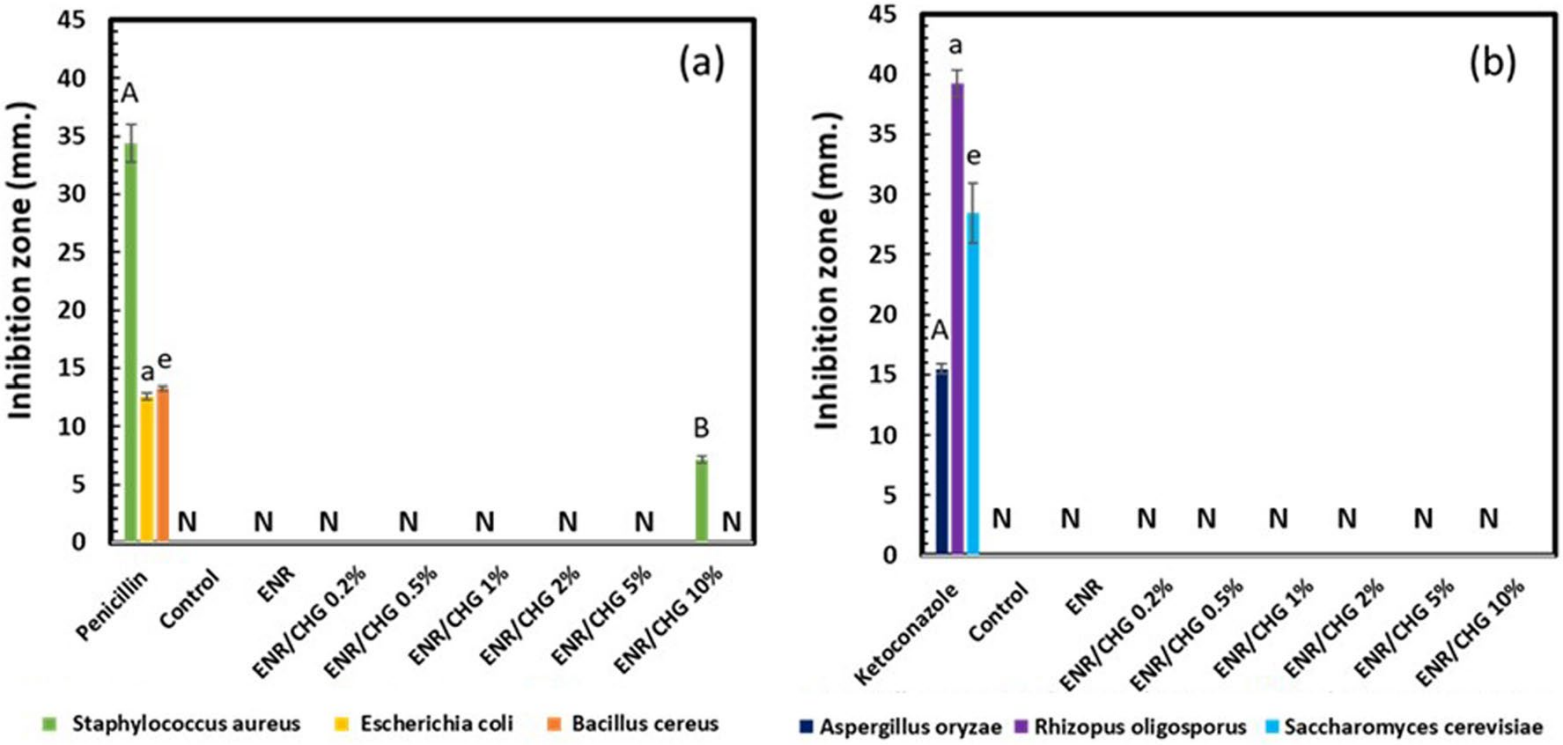
Inhibition zones of ENR and ENR blends with 0.2, 0.5, 1, 2, 5, and 10% (w/w) CHG: (**a**) bacteria and (**b**) fungi; *n* = 3. N indicates not detected. The mean values indicated by A‒C uppercase letters (*Staphylococcus aureus* and *Aspergillus oryzae*), a‒c lowercase letters (*Escherichia coli* and *Rhizopus oligosporus*), and e‒g lowercase letters (*Bacillus cereus* and *Saccharomyces cerevisiae*) significantly differ (*P* < 0.05).

**Figure 9:**
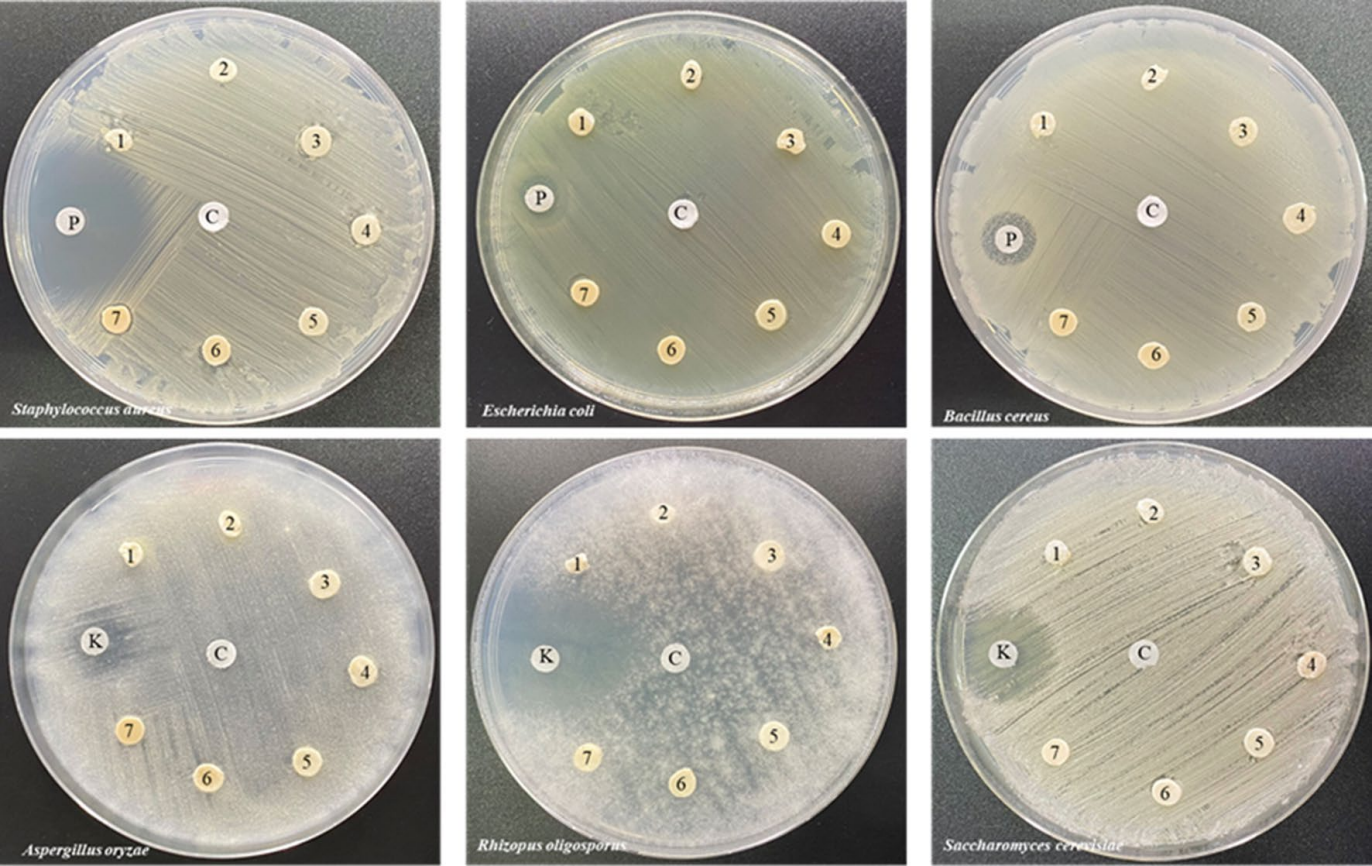
Inhibition zone images of (1) ENR and ENR blends with (2) 0.2%, (3) 0.5%, (4) 1%, (5) 2%, (6) 5%, and (7) 10% CHG, (P) positive control with penicillin, (K) positive control with ketoconazole, and (C) negative control on the microbial activity against *Staphylococcus aureus*, *Escherichia coli*, *Bacillus cereus*, *Aspergillus oryzae*, *Rhizopus oligosporus*, and *Saccharomyces cerevisiae*.

In vitro antimicrobial activity was assessed by examining the inhibition zones of the growth area, as shown in Fig. 8. Penicillin and ketoconazole were used as the antibacterial and antifungal positive controls, respectively. Penicillin shows inhibition zones for three bacterial species (Fig. 8a): *Staphylococcus aureus* (gram-positive bacteria), *Escherichia coli* (gram-negative bacteria), and *Bacillus cereus* (gram-positive bacteria), whereas ketoconazole exhibits inhibition zones for three fungi (Fig. 8b): *Aspergillus oryzae*, *Rhizopus oligosporus*, and *Saccharomyces cerevisiae*. No inhibition zones were observed for any microbial species treated with ENR, whereas ENR/CHG10 presented an inhibition zone against *Staphylococcus aureus.* Low CHG reacted with ENR and connected with ENR structure. CHG 10% performed high reaction with ENR, while excessive amount of free CHG was remained inside ENR structure. Thus, the addition of 10% CHG inhibited *Staphylococcus aureus* because the network structure formed due to the reaction between CHG and ENR which trapped the excessive amount of free CHG. The other ENR/CHG blends showed no inhibitory effect because they were not retained free CHG. This was owing to the rubber modification performance of the CHG reaction, which remained inside the ENR structure, resulting in poor antimicrobial delivery. The network structure of ENR/CHG10 encapsulated free CHG, which inhibited *Staphylococcus aureus.* The images of the inhibition zones are shown in Fig. 9.

## Conclusions

The mechanical and antimicrobial properties of ENR were successfully improved via blending with CHG. The new FTIR peak at 1746 cm^−1^ confirmed the reaction between the epoxy groups of ENR and the amino groups of CHG. Blending with CHG improved the maximum tensile strength of ENR from 50 kPa for pure ENR to 190 kPa for the ENR with 10% CHG. The addition of CHG to ENR improved the mechanical and elastic properties. The hardness of the ENR/CHG blend increased with CHG content because of the crosslinking reaction with the CHG structure. Swelling ratios of the specimens were not significantly differ owing to ENR polarity. Morphology studies showed good compatibility between ENR and CHG owing to their miscibility in the blend. In the antimicrobial activity tests, the blend with 10% CHG showed an inhibitory effect on *Staphylococcus aureus*. Thus, the reaction between the NH_2_ groups of CHG and the epoxy groups of ENR improved the mechanical, elastic recovery, and antimicrobial properties of the blends. The ENR/CHG blend can be applied as a rubber in medical and cushioning applications.

## Data Availability

The datasets used and/or analysed during the current study available from the corresponding author on reasonable request.
